# In vivo Exposure Effects of ^99m^Tc-methoxyisobutylisonitrile on the *FDXR* and *XPA* Genes Expression in Human Peripheral Blood Lymphocytes

**DOI:** 10.22038/aojnmb.2017.9678

**Published:** 2018

**Authors:** Mohammad Taghi Bahreyni-Toossi, Habibeh Vosoughi, Hosein Azimian, Abdul Rahim Rezaei, Mehdi Momennezhad

**Affiliations:** 1 Medical Physics Research Center, Mashhad University of Medical Sciences, Mashhad, Iran; 2 Immunology Research Center, School of Medicine, Mashhad University of Medical Sciences, Mashhad, Iran; 3 Nuclear Medicine Research Center, Mashhad University of Medical Sciences, Mashhad, Iran

**Keywords:** Biodosimetry, DNA damage, Gene expression, Human peripheral blood lymphocytes, Ionizing radiation

## Abstract

**Objective(s):**

In recent years, the application of radiopharmaceuticals in nuclear medicine has increased substantially. Following the diagnostic procedures performed in nuclear medicine departments, such as myocardial perfusion imaging, patients generally receive considerable doses of radiation. Normally, radiation-induced DNA damages are expected following exposure to a low-dose ionizing radiation. In order to detect molecular changes, high-sensitivity techniques must be utilized. The aim of this study was to assess the effect of a low-dose (below 10 mSv) gamma ray on gene expression using quantitative real-time polymerase chain reaction (qRT-PCR).

**Methods:**

Blood samples were obtained from 20 volunteer patients who underwent myocardial perfusion imaging. They were given various doses of Technetium-99m methoxyisobutylisonitrile (^99m^Tc-MIBI). After that, peripheral blood mononuclear cells (PBMNs) were derived, and then total RNA was extracted and reverse-transcribed to cDNA. Finally, the expression levels of xeroderma pigmentosum complementation group-A *(XPA)* and ferredoxin reductase *(FDXR)* genes were determinded through qRT-PCR technique using SYBR Green.

**Results:**

*XPA* and *FDXR* expression levels were obtained following a very low-dose ionizing radiation. A significant up-regulation of both genes was observed, and the gene expression level of each individual patient was different. If differences in the administered activity and radiosensitivity are taken into account, the observed differences could be justified. Furthermore, gender and age did not play a significant role in the expression levels of the genes under study.

**Conclusion:**

The up-regulation of *FDXR* after irradiation revealed the high-sensitivity level of this gene; therefore, it could be used as an appropriate biomarker for biological dosimetry. On the other hand, the up-regulation of *XPA* is an indication of DNA repair following radiation exposure. According to linear no-threshold model (LNT) and the results obtained from this study, a very low dose of ionizing radiation could bring about adverse biological effects at molecular level in the irradiated person.

## Introduction

All living creatures are exposed to natural sources of ionizing radiation during their lifetime. Natural radiation emanates from soil, synthetic materials, foods, drinks, cosmic rays, and internal radioactive sources (1, 2).

Since the discovery of X-ray and radioactive materials, exposure to man-made sources, especially medical ones, has increased. Both natural and man-made sources of ionizing radiation contribute to human exposure and consequently pose a possible risk to human health. A part of this issue is unavoidable, for example the natural background radiation. As the application of man-made sources expanded, so did the potential health risks and the public concerns (2). In recent years, the general population have received considerable doses of such radiations from diagnostic medical procedures such as nuclear medicine imaging, interventional fluoroscopy, and computed tomography (CT) (3). Therefore, it is important to investigate the potential health risks arising from high-dose diagnostic procedures.

Moreover, the number of cardiac diagnostic procedures involving the use of ionizing radiation has increased rapidly (4). Technetium-99m methoxyisobutylisonitrile (^99m^Tc-MIBI) is used for myocardial perfusion imaging and ^99m^Tc is obtained from ^99m^Mo in a generator. The physical half-time of ^99m^Tc is 6 hours and its biological half-time is 1 day (in terms of human activity and metabolism); it emits 140.5 KeV gamma rays. These characteristics make the isotope suitable for diagnostic scanning procedures. If ^99m^Tc is used as a radiotracer and indicator of coronary blood flow, it must be bound to sestamibi or tetrofosmin and concentrated in the myocardium (5). ^99m^Tc is taken up not only by myocardium, but also by the highly radiosensitive cells like human peripheral blood lymphocytes (6). The effective dose for most nuclear medicine procedures varies approximately from 0.3 to 20 mSv (3). Therefore, diagnostic nuclear medicine procedures are considered as low-dose examinations (5-100 mGy). Beir VII concluded that the available biological and biophysical data support a linear no-threshold (LNT) risk model for the low-dose radiation. According to this model, even the lowest dose of radiation may be a potential risk for human health (2).

The induced harmful biological effects in a cell after exposure to ionizing radiation is caused primarily by damages to DNA molecules (7). Upon exposure to radiation, cells normally react in three distinct ways, namely arresting cell cycle progression, repairing DNA lesions, or triggering the apoptosis response (8). Classical genetic analysis techniques were employed to identify an ever-expanding number of genes activated directly or indirectly by DNA repair, cell cycle progression, and apoptosis (9). DNA damage is investigated by cytogenetic techniques such as detection of chromosome abnormality or molecular techniques such as quantitative real-time PCR (qRT-PCR) (1).

In the current study, we sought to assess the effect of low-dose (below 10 mSv) gamma ray (^99m^Tc-MIBI) on the expression of Xeroderma pigmentosum complementation group-A (*XPA*) and ferredoxin reductase (*FDXR*) genes in human peripheral blood lymphocytes using qRT-PCR.

## Methods

### Myocardial perfusion imaging procedure

In this study, patients underwent myocardial perfusion single-photon emission computed tomography (SPECT) with a 2-day protocol. A pharmacologic stress test was carried out with infusion of dipyridamole during 4 min. When the pharmacological effect reached the maximum level, 740 MBq of ^99m^Tc-MIBI was injected intravenously, and 90 min later, stress gated imaging was performed in the supine/prone position. Rest images were obtained on the next day (24 h later), and the same technique was replicated.

### Collection of blood samples

The participants included 20 volunteer patients (15 females, 5 males; mean age 55±11.68 years) who were referred to the Department of Nuclear Medicine in Ghaem Hospital of Mashhad, Iran for myocardial perfusion imaging. Patients with no history of radiation therapy, occupational exposure, genetic diseases, and smoking were entered into the study. Furthermore, a questionnaire was filled in for every patient in order to collect their history of medical and professional exposure to radiation.

We collected 3 ml whole blood samples in individual tubes containing ethylene diamine tetra-acetic acid (EDTA). The blood samples were obtained before the injection of ^99m^Tc-MIBI (as the control) and 24 h after the first injection of the radiopharmaceutical. The patients received a 20-30 mCi dose of ^99m^Tc-MIBI, according to their weights.

### PBMNs separation and RNA extraction

PBMNs were isolated from the whole blood samples by density-gradient centrifugation using Ficoll (Cedarlane Labs, Canada) according to the manufacturer’s instructions, and then the lymphocytes were washed with phosphate-buffered saline (PBS). Immediately, TriPure reagent (Roche Applied Science, Germany) was utilized for isolation of total RNA, the same way as described in our previous study (10). In addition, to check the quality and purity of the RNA samples, 2% agarose gel electrophoresis was used.

### cDNA synthesis

First-strand cDNA synthesis was performed using 1 µg of total RNA utilizing the RevertAid ™ First Strand cDNA Synthesis Kit (Fermentas, Germany) according to the manufacturer’s recommendations.

### Real time PCR

Different primer sets were designed using Beacon Designer software, version 7 (PREMIER Biosoft International, Palo Alto, CA, USA), and their specificity was checked using BLAST analysis (NCBI, USA). For validation of the primers, amplification of a single product for each primer set was confirmed by endpoint PCR followed by DNA sequencing (Applied Biosystems, SEQLAB, Germany). The sequences of primers are presented in Table 1. The RT-PCR, using Syber Green method, was carried out on cDNA samples with the SYBR^®^ Premix Ex Taq™ (Takara; Otsu Shiga, Japan) for *XPA* and *FDXR*. Beta-2 microglobulin (*β2M*) was applied as the reference gene to adjust and control the error in mRNA expression among the samples. In order to quantify the expression of each gene, five standards were prepared using 10-fold serial dilution of a concentrated standard sample for the gene of interest and the reference gene. RT-PCR was carried out in a MicroAmp^®^ Fast Optical 48-well Reaction Plate (applied Biosystems, USA) with Microamp™Optical Adhesive Film (applied Biosystems, USA). The mixture in each well contained 7.5 µl of SYBR^®^ premix Ex Taq™ II (2x), 5.1 µl of dH_2_O, 0.3 µl of ROX™ Reference Dye II (50x), 0.03 nM of forward and reverse primers, and 1.5 µl of diluted cDNA. The primer sequences are presented in Table 1.

**Table 1:** The primer sequences

Gene	Primer sequence (5’ to 3’)
*B2M*	Forward: GTATGCCTGCCGTGTGAAC
	Reverse: AACCTCCATGATGCTGCTTAC
*FDXR*	Forward: CATAGCCACAACCATGACTGACAG
	Reverse: CCACCTCCTCGGCATCCA
*XPA*	Forward: CTGGAGGCATGGCTAATG
	Reverse: CAAATTCCATAACAGGTC

The RT-PCR cycling included initial denaturation at 95° c for 10 min, followed by 40 cycles at 95° c for 10 s and at 60° c for 30 s. Relative standard curve method was applied for cDNA quantification. This approach provided highly accurate quantitative results. A melt curve analysis was also performed to verify if single products were produced. Finally, the relative quantity of the gene of interest was normalized to the relative quantity of *β2M* as the reference gene, and the relative expression of *XPA* and *FDXR* genes were determined, as well.

### Statistical analysis

Wilcoxon matched-pair test and t-test were performed to compare the groups exposed to gamma rays of ^99m^Tc before and after ^99m^Tc-MIBI injection.

## Results

### Measurement of the received doses by the patients

It should be noted that the injected activity is always a compromise between the highest activity to obtain the best image quality and the lowest activity to keep the radiation doses as low as possible (11). Therefore, the administered activity determined the received dose per kilogram of body weight, which accordingly increased for obese patients (11, 12). The administered activity was considered approximately 25 mCi/70 kg body weight as the standard (4, 13). According to the International Commission on Radiological Protection (ICRP) publication 80, the effective dose for Cardiac rest-stress test (^99m^Tc-sestamibi 2-day protocol) is 0.0085 mSv/MBq (0.0079 stress, 0.0090 rest) (3). The cumulative radiation dose is calculated by the equation suggested in Medical Internal Radiation Dose (MIRD) pamphlets:


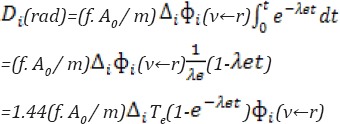


Based on the above equation, the absorbed dose is proportional to A_0_ (initial administered activity) per body weight (Kg) (14). In the present study, A_0_/m and the effective absorbed dose for individual patients were roughly calculated. Patient’s data are presented in Table 2.

**Table 2:** Patients’ data and received doses

	Gender	Age (year)	A_0/m_	Effective dose (mSv)
**1**	Female	75	0.350	5.7313
**2**	Female	39	0.243	3.9792
**3**	Female	52	0.266	4.3558
**4**	Female	60	0.247	4.2469
**5**	Female	57	0.333	5.7256
**6**	Male	52	0.261	4.7013
**7**	Female	59	0.275	4.9535
**8**	Male	44	0.288	6.1309
**9**	Female	70	0.350	6.0179
**10**	Female	40	0.225	3.8686
**11**	Female	30	0.326	8.0075
**12**	Female	52	0.323	5.5537
**13**	Female	52	0.294	4.8143
**14**	Female	62	0.303	4.9617
**15**	Male	56	0.285	4.6669
**16**	Female	79	0.466	8.0124
**17**	Female	57	0.294	4.8143
**18**	Female	53	0.268	4.8274
**19**	Male	53	0.268	4.8274
**20**	Male	60	0.370	6.0588

The received dose by the patients was within the range of 3.8686 to 8.0124 mSv. The median and mean of the doses were measured to be 4.8904 mSv and 5.3127 mSv, respectively.

### Assessment of the gene expression level

**Figure 1:**
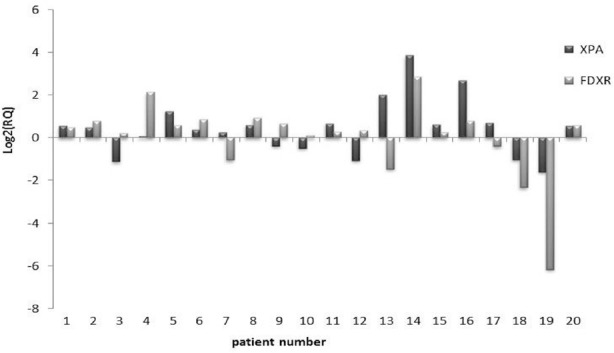
Illustration of relative expression levels of XPA and FDXR genes for each patient following irradiation; when Log_2_ (RQ) is used, gene expression level is better demonstrated (31)

The gene expression level was estimated for each individual patient separately. The expression levels of *XPA* and *FDXR* genes are exhibited in Figure 1. We observed up-regulation of *XPA* in 13 patients and down-regulation of *XPA* in 7 patients. Up-regulation of *FDXR* in 14 patients and down-regulation in 6 patients were also noticed. We did not recognize any specific patterns of gene expression in patients. However, in most cases, *XPA* and *FDXR* genes were up-regulated.

The results obtained by RT-PCR are relative quantifications (RQ). In interpretation of the gene expression levels, RQ > 1 represents up-regulation and 0 < RQ < 1 indicates down-regulation. In the present study, log_2_ (RQ) was used to measure the relative expression. Up-regulations were Log_2_ (RQ) transformed to positive values and down-regulations were transformed to negative values.

The expression levels of the two selected genes following irradiation by ^99m^Tc gamma ray were different for each individual patient. Furthermore, we noted that gender and age played a role in the expression of the studied genes. Previous studies also indicated the effect of gender, age, diet, and life style on DNA damage (15, 16).

### Gene expression

**Figure 2:**
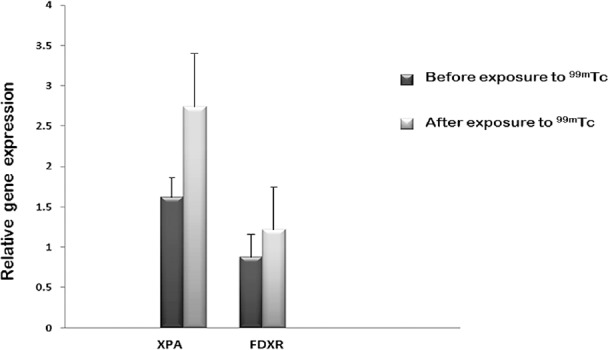
Relative expression levels of XPA and FDXR genes before and 24 h after exposure to gamma ray of ^99m^Tc; up-regulation of XPA and FDXR levels was observed in human peripheral blood lymphocytes

The results demonstrated a significant elevation in the expression level of *FDXR* gene (P=0.05) and a non-significant increase in *XPA* gene (P=0.1). The relative gene expressions before and after exposure to ^99m^Tc gamma ray are shown in Figure 2. It seems that by expanding the number of samples, p-values reached significant levels.

### Effect of gender and age

**Figure 3:**
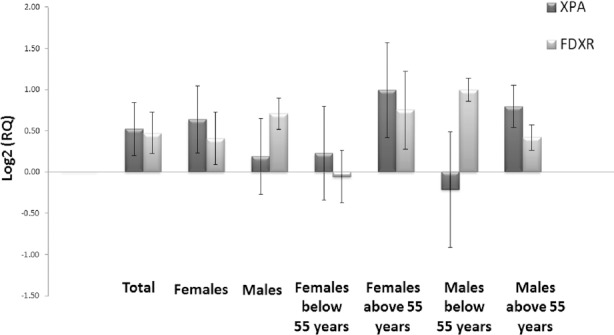
Relative expression of XPA and FDXR genes following irradiation considering patients’ gender and age; when Log_2_ (RQ) is used, gene expression level is better demonstrated

With regard to the mean age, there were two groups of patients below and above 55 years old in both males and females separately. There was no significant difference between the two age groups in the expression levels of the selected genes (P-value for *XPA*=0.18 and for *FXDR*=0.3). Furthermore, the expression level of *FDXR* and *XPA* for male and female patients were not different significantly (P-value for *XPA*=0.5 and for *FDXR*=0.6). Only non-significant differences between males above 55 years old and below 55 years old (P-value for *XPA*=0.3 and for *FDXR*=0.1) and females above 55 years old and below 55 years old (P-value for *XPA*=0.3and for *FDXR*=0.2) were observed. As the patients were divided according to their gender and age, the expression levels of both genes increased insignificantly (Figure 3).

## Discussion

In recent years, the application of nuclear medicine procedures as a diagnostic technique has increased sharply. The biological effects of the administered radiopharmaceuticals at molecular levels have been investigated by cytogenetic and chromosome aberration tests. Although some results were obtained through accredited methods, approaches with higher accuracy and sensitivity are still required. Recently, molecular techniques have been increasingly utilized and it seems that gene expression is an authentic process for genetic and biological studies. Development of gene expression is an appropriate method to assess the radiation sensitivity in an exposed person. Through gene expression, the researcher is able to estimate and report the dose (within a range of 0.3 to 0.5 Gy) more accurately and quickly compared to other laboratory tests used for dose estimation (17).

Most studies on gene expression and biological dosimetry were performed in low and high levels of exposure (10, 17, 18), and few of them discussed very low doses of ionizing radiation (18). In this study, variation of the gene expression level was measured by RT-PCR technique in order to assess the biological effect of in vivo irradiation of very low-dose gamma rays of ^99m^Tc-MIBI.

One cellular response to DNA damage is the alteration of gene expression. Another response to DNA damage induced by ionizing radiation is the activation of repair mechanisms. Wood specified 130 known human DNA repair genes, one of which was *XPA* (19). *XPA* gene is associated with the nucleotide excision repair (NER) pathway. NER is able to remove bulky helix-distorting DNA lesions caused by physical and chemical carcinogens. *XPA* plays a critical role in positioning the repair machinery precisely around the lesions. If the repair of DNA lesion fails, some P53 target genes such as *FDXR* are up-regulated in the apoptosis pathway (20).

The genome is protected from oxidation by endogenous reactive oxygen species (ROS) through the tumor suppressor p53 (1). The increase in ROS levels is attributed to apoptosis induction. ROS are formed as a natural byproduct of respiration during the transfer of electrons in the mitochondria. In the mitochondria, *FDXR* transfers electrons from nicotinamide adenine dinucleotide phosphate (NADPH) to cytochrome P450 during oxidative phosphorylation. *FDXR* is an essential target gene for the p53 family that is induced by DNA damage and plays a pivotal role in apoptosis induction (21).

Fachin et al. used cDNA microarray technique to demonstrate the gene expression profiles in radiation workers exposed to chronic low-level radiation. In their study, alterations in 78 genes including *XPA* (as a DNA repair gene) were statistically significant. Their results also showed that *XPA* can be specified as a biomarker in chronic low-dose exposure (22). It is now possible to estimate the radiation dose after a specified time following exposure by a single biomarker (a single gene expression), which is a significant advantage for Biodosimetry (23). *FDXR* is a target gene that is up-regulated 24 h after irradiation (24) and is known as a highly sensitive gene to low and high doses of ionizing radiation (1, 25). Paul et al. concluded that *FDXR* is an inherent responsive gene 24 and 48 h after exposure to low doses of ^137^Cs; therefore, it may be applied for the prediction of low doses in biological dosimetry (25). Manning et al. also demonstrated the up-regulation of *FDXR* gene 24 h after exposure at doses as low as 10 mGy. A linear regression between the gene expression level and the absorbed dose was obtained that could be used for dose estimation (1).

The expression levels of the two genes were different for each patient even with the same effective dose of ionizing radiation. These results may reflect the inter-individual variability of the donors as indicated in our previous study (26). The development of gene expression is an appropriate method to assess the radiation sensitivity in an exposed person. It also provides information with regards to the probability of occurrence of long-term effects such as carcinogenesis (1).

In the present study, we performed in vivo irradiation of lymphocytes, and experimental arrangements were actually similar to the real-life conditions. The patients were selected for a specific imaging procedure and received the same radiation dose through radiopharmaceutical injection. Nevertheless, the observed response to the radiation exposure, which varied for each patient, is hinged upon the individual’s lifestyle and radiosensitivity. With respect to patients’ dose (measured approximately), patients no. 13 and 14 could be classified as radiosensitive and patient no. 11 as a radio-resistant since the expression levels of the two genes increased for radiosensitive patients and decreased for radio-resistant ones.

The results of this study revealed high expression levels of both genes in patients exposed to very low doses of ionizing radiation in in vivo conditions. Fachin et al. used cDNA microarray technique to demonstrate the gene expression profiles in radiation workers. In their study, alterations in 78 genes including *XPA* were statistically significant. The results of this study also showed that *XPA* can be specified as a biomarker in chronic low-dose exposure (22). The absorbed dose of in vivo irradiation was acquired from a range of very low doses of ionizing radiation. No significant up-regulation of *XPA* was observed in their study. In order to achieve a higher accuracy (smaller P-values), blood samples from a larger number of donors must be examined. Our results were not consistent with the findings of Fachin et al. The up-regulation of *XPA* is an indication of DNA repair following the exposure, which is confirmed by the chronic low-dose exposure of ionizing radiation.

*FDXR* is a target gene that is up-regulated 24 h after irradiation. It is known as a highly sensitive gene to low and high doses of ionizing radiation (1, 25). Paul et al. demonstrated that *FDXR* is an inherent responsive gene 24 and 48 h after exposure to low doses of ^137^Cs; therefore, it may be used for the prediction of low doses in biological dosimetry (25). Manning et al. demonstrated up-regulation of *FDXR* gene 24 h after exposure at doses as low as 10 mGy. A linear regression between gene expression level and absorbed dose was obtained, which may be utilized for dose estimation (1). The results of our study were in good agreement with those of the abovementioned studies (1, 23, 25). We demonstrated a significant induction of *FDXR* gene up-regulation through in vivo irradiation of human peripheral blood lymphocytes; therefore, *FDXR* gene has the potential to be used as a biomarker for estimation of absorbed doses from the uptake of radioactive sources in our body.

The relative gene expressions were compared in blood samples of male and female donors in different age groups; however, no significant differences were observed between males and females or the two age groups. These findings were consistent with the results of Vodicka et al. that examined DNA repair system in healthy donors. They showed that DNA repair did not depend on the age of donors, and there were no significant variations in the samples obtained from either of the genders (27). Additionally, Manning et al. demonstrated that the expression level of *FDXR* gene was independent of gender and age (1).

Several researchers also ascribed that clinical doses of ^99m^Tc-MIBI could not induce significant DNA lesions or genetic damages (28, 29). In one study, chromosomal aberrations were used to estimate the very low doses absorbed due to exposure to ^99m^Tc in nuclear medicine. Also, the chromosomal damage did not deteriorate 24 h after ^99m^Tc-MIBI injection (29). In another study, the researchers found no increase in the frequency of induced micronuclei in lymphocytes following the exposure to low doses of ^99m^Tc-MIBI (28).

Molecular techniques such as RT-PCR are more accurate and sensitive compared to techniques such as micronuclei or cytogenetic assays that were utilized in the previous studies. Earlier findings demonstrated a significant increase in the gene expression, implying that DNA damage could occur with a very low dose of ^99m^Tc-MIBI. Our results also revealed the genetic hazardous effects of in vivo irradiation of ^99m^Tc-MIBI in peripheral blood lymphocytes. In addition, the effects of ^99m^Tc-MIBI on the possibility of chromosomal aberrations in human peripheral blood lymphocytes were studied through in vitro exposure to ^99m^Tc. At doses above 10 cGy, an increase in the frequency of induced micronuclei and apoptosis induction was observed (6). The results also showed the up-regulation of *FDXR* gene following the irradiation by ^99m^Tc-MIBI gamma ray. Moreover, apoptosis was observed following a very low-dose exposure (below 10 mSv).

The limitations of our study included small sample size and inaccurate measurement of doses of patients that were irradiated by ^99m^Tc gamma rays. Internal dosimetry is an important challenge in nuclear medicine studies and MIRD formula is used for calculation of the received dose in patients. Most of the studies on biodosimetry were carried out on samples irradiated by external sources of ionizing radiation in which the received doses were clear; however, in internal radiation, the exact amounts of the received doses in patients were unknown.

## Conclusion

Clinical doses that are normally used in nuclear medicine procedures are within the low-dose range (below 100 mSv) (3, 30). Therefore, assessing the biological damages in the irradiated cells demands a sensitive molecular technique like gene expression profiling using RT-PCR. In our study, the alteration in expression levels of *XPA* and *FDXR* genes was studied in irradiated lymphocytes that were exposed to in vivo ^99m^Tc gamma rays. Our results revealed the DNA damages and confirmed LNT for dose response, which implies that even the lowest dose of ionizing radiation could cause genetic effects.
